# Supplementary material for: Structural basis for the mechanisms of human presequence protease conformational switch and substrate recognition
Source: Nat Commun. 2022 Apr 5;13:1833. doi: 10.1038/s41467-022-29322-4 (PMC8983764; doi:10.1038/s41467-022-29322-4)
Supplement: Supplementary file 1 — Supplementary Information [file 41467_2022_29322_MOESM1_ESM.pdf]

Supplementary Information for

"Structural basis for the mechanisms of human presequence protease conformational switch and substrate recognition"

Wenguang G. Liang[1], Juwina Wijaya[2], Hui Wei[3], Alex J. Noble[3], Jordan M. Mancl[1], Swansea Mo[1], David Lee[4], John V. Lin King[5], Man Pan[6], Chang Liu[6], Carla M. Koehler[2], Minglei Zhao[6], Clinton S. Potter[3], Bridget Carragher[3], Sheng Li[4], Wei-Jen Tang[1,*]

Nature Communications 2022

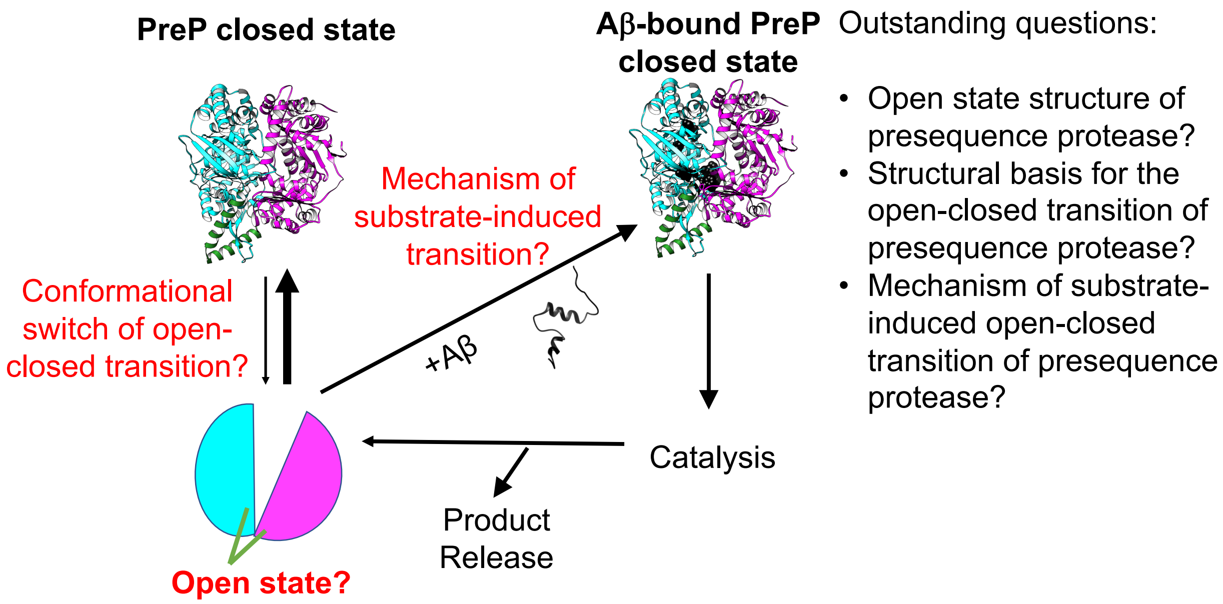

Supplementary Figure 1 Summary of key unsolved PreP structures. Schematic diagram for the catalytic cycle of PreP. Prior to this report, only the closed state structures of presequence protease and the related M16C metalloproteases have been reported. In the closed conformation, substrate cannot enter into the catalytic chamber while the proteolytic products cannot be released. The open-closed transition of PreP is postulated to be mediated by the rigid body motion between PreP-N and PreP-C. In the absence of substrate, PreP is favored in the closed state. Until now, the structure of open state PreP has not been reported. Thus, the structural basis for the open-closed transition of PreP remains to be determined. As the crystal structures of PreP in the presence or absence of Aβ are near identical, precise structural differences between substrate-bound and apo-PreP and mechanism for substrate-induced open to closed transition remains to be determined.

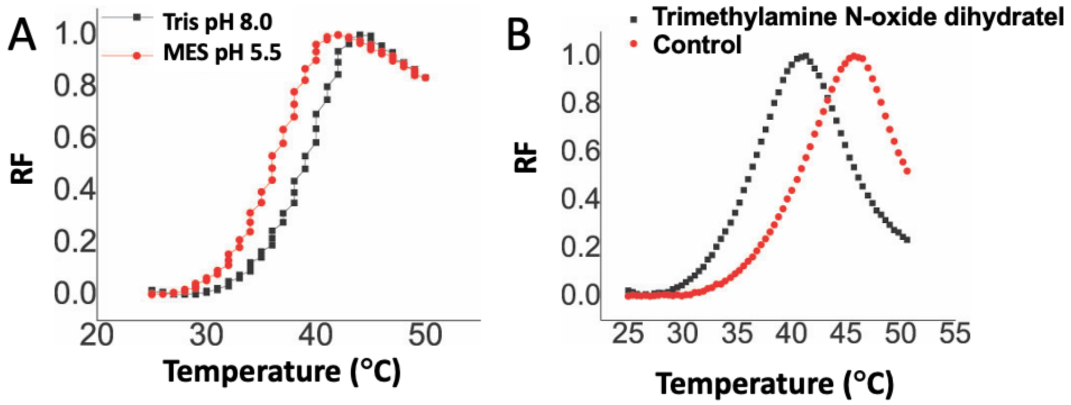

Supplementary Figure 2 Differential scanning fluorimetry of PreP. Exemplary profiles on the effect of (A) buffers and (B) additives on the change of thermal denaturation profiles. 40 buffer conditions and 98 additives were screened. The control buffer is Tris, pH 8.0

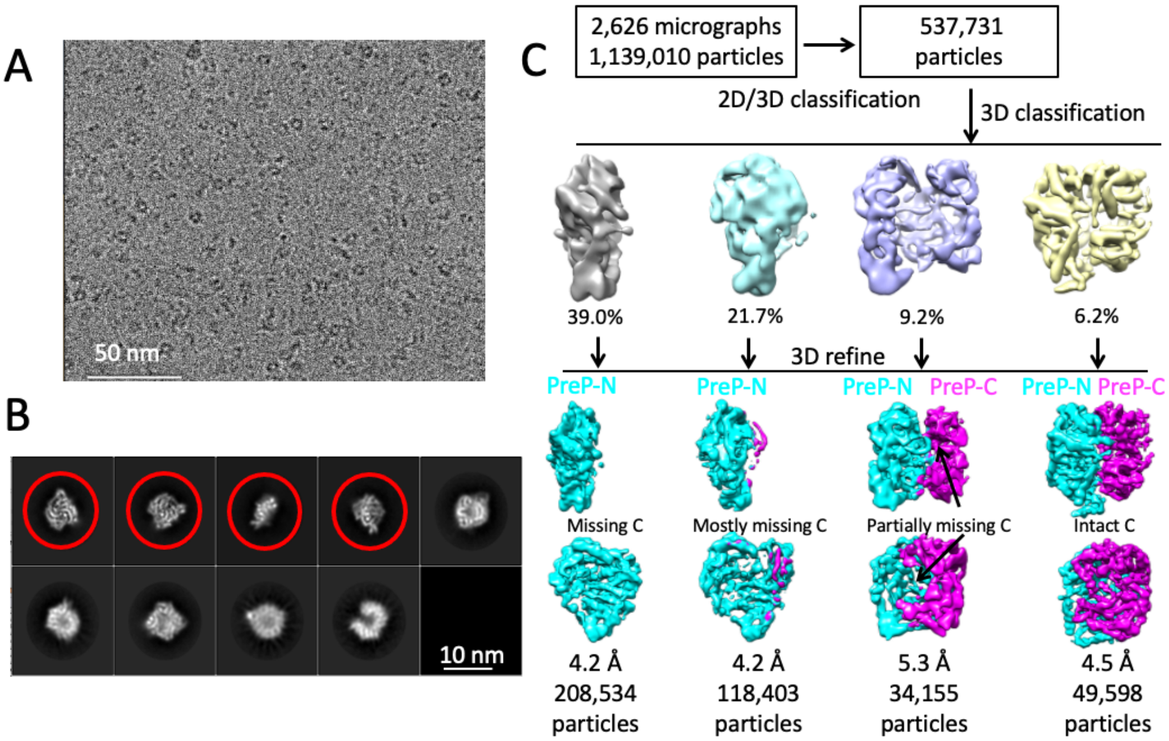

Supplementary Figure 3 CryoEM analysis of apo-PreP using grid prepared by vitrobot. (A) CryoEM micrograph for the apo-PreP dataset representative of the 2,626 micrographs collected. (B) 2D classification of PreP. The 2D classes that contain the PreP-N domain and the denatured PreP-C domain are highlighted by red circles. (C) The workflow of 2D/3D classification.

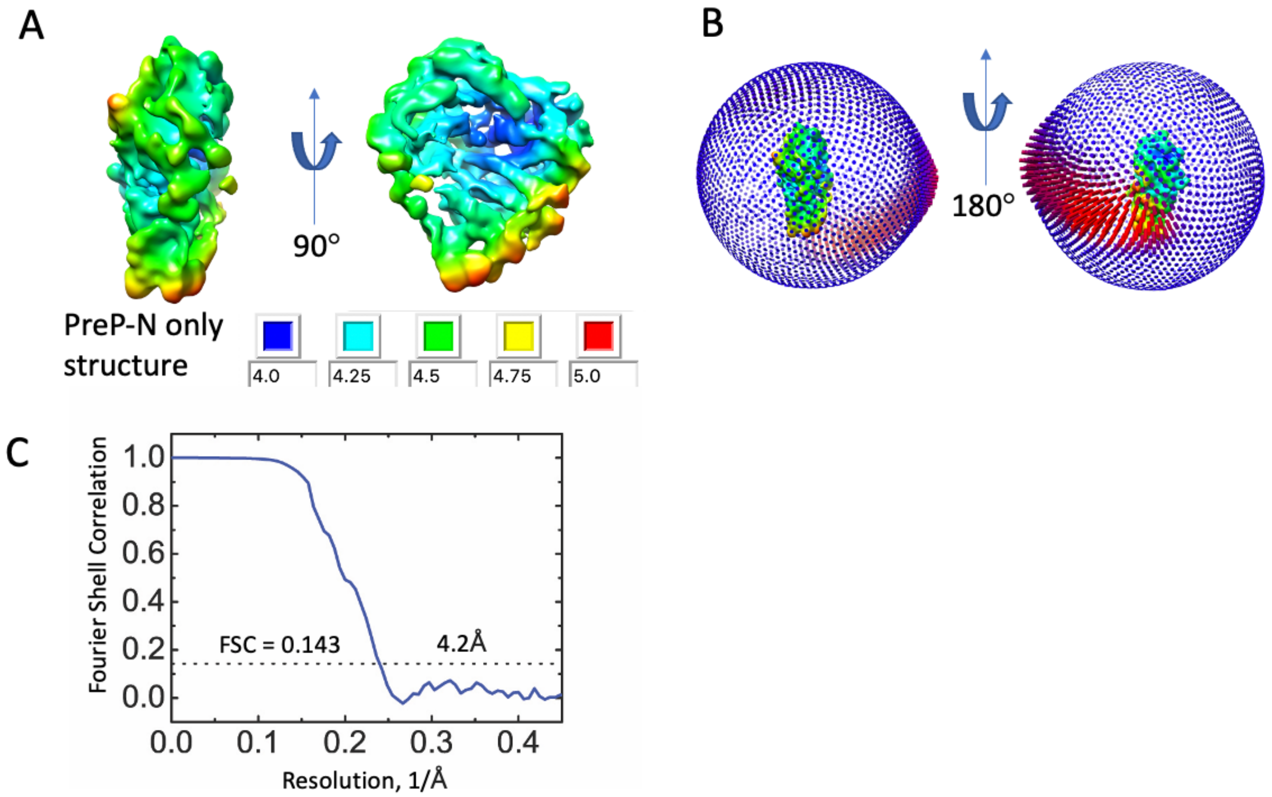

Supplementary Figure 4 Cryo-EM statistics of PreP that has intact PreP-N and denatured PreP-C using the grid prepared by vitrobot. (A) Final refined map colored according to local resolution generated from RELION3.0. (B) Angular distribution plots generated from RELION3.0. (C) FSC plot and resolution estimation using gold-standard 0.143 criterion generated from RELION3.0.

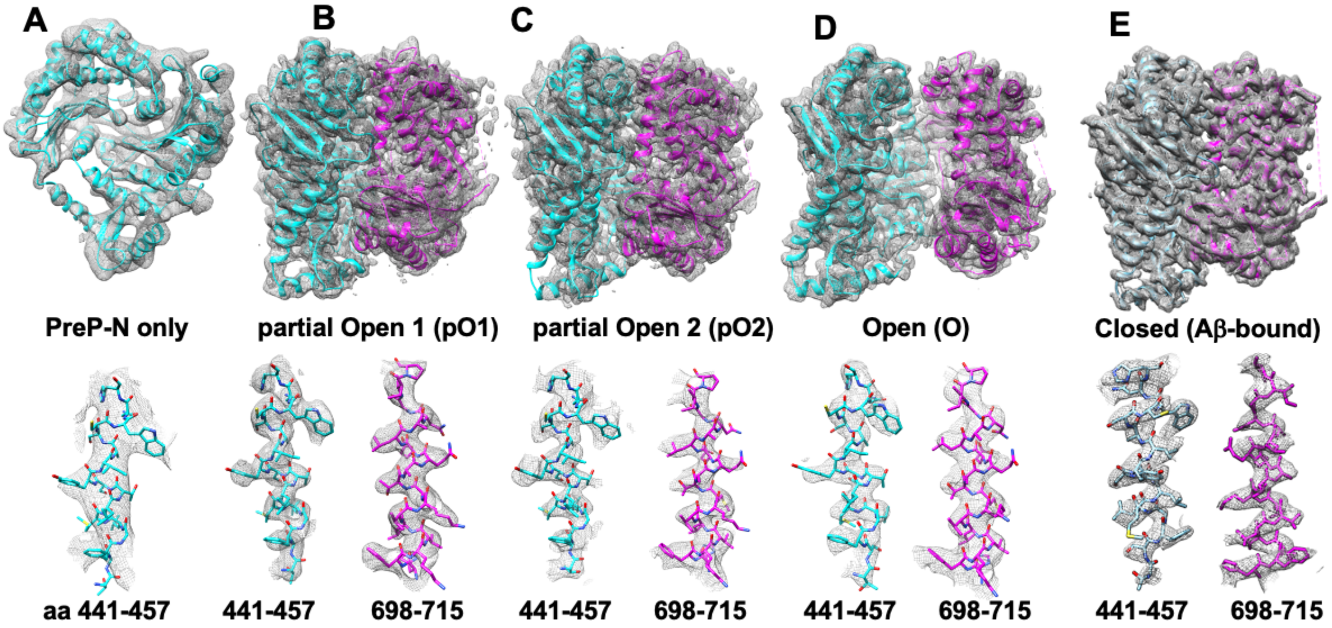

Supplementary Figure 5 Summary of the solved structures and their map quality. (A) PreP particles with denatured C-terminal region from conventional blotting. (B-D) partial open and open states of Apo-PreP from Chameleon prepared grids. (E) Substrate bound closed state of PreP solved in the presence of amyloid beta.

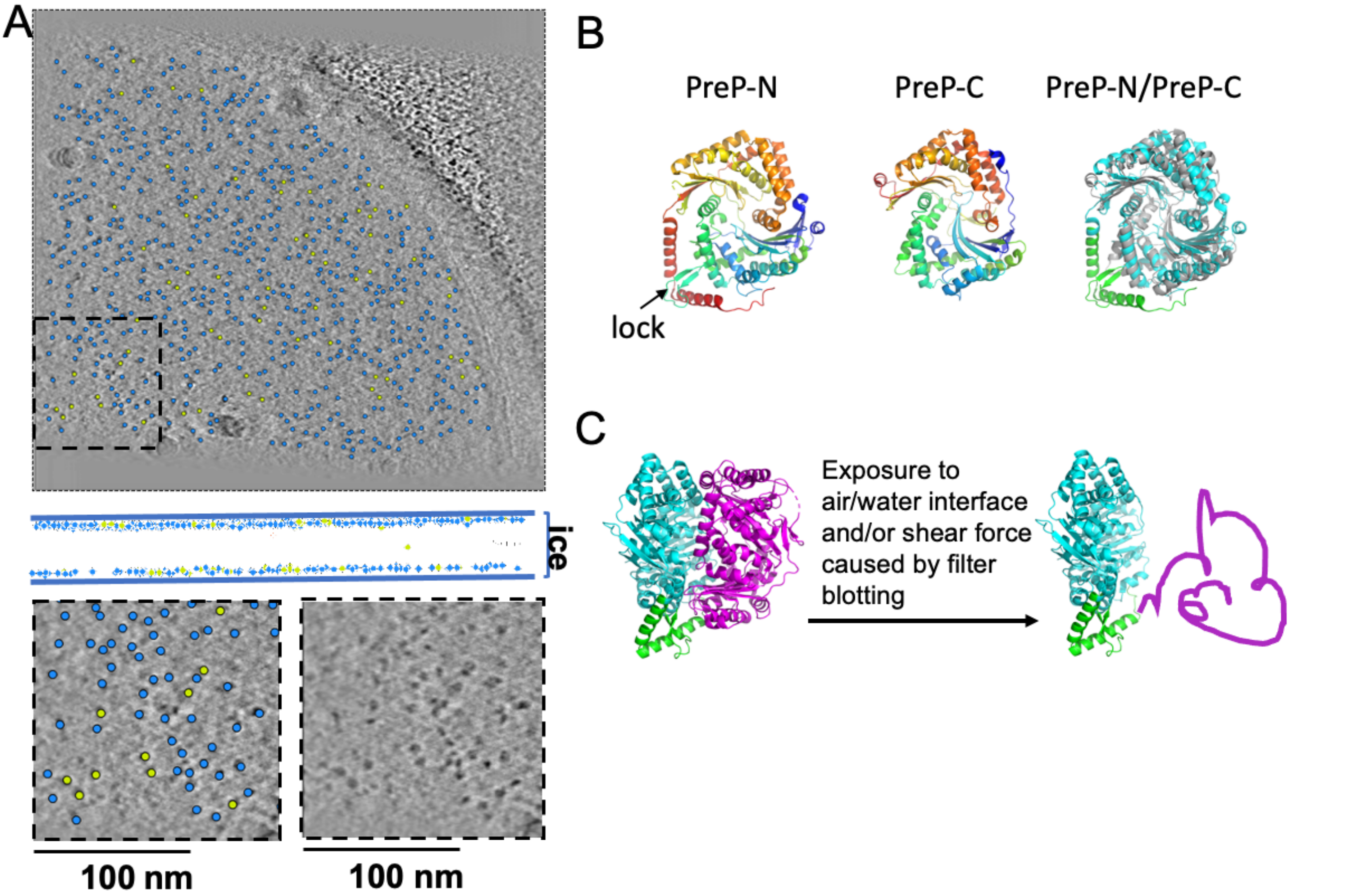

Supplementary Figure 6 Molecular basis for the preferential denaturation of PreP C-terminal domain when vitrobot was used for vitrification. (A) CryoET analysis of vitrobot prepared grid of PreP. Slices of tomograms showing particles distribution, ~12% of particles are full particles (yellow) and ~88% are denatured half particles (blue). (B) PreP domain architecture. (C) Hypothesis for the observed PreP half particles. PreP N shown in cyan, PreP C in pink, linker region in green.

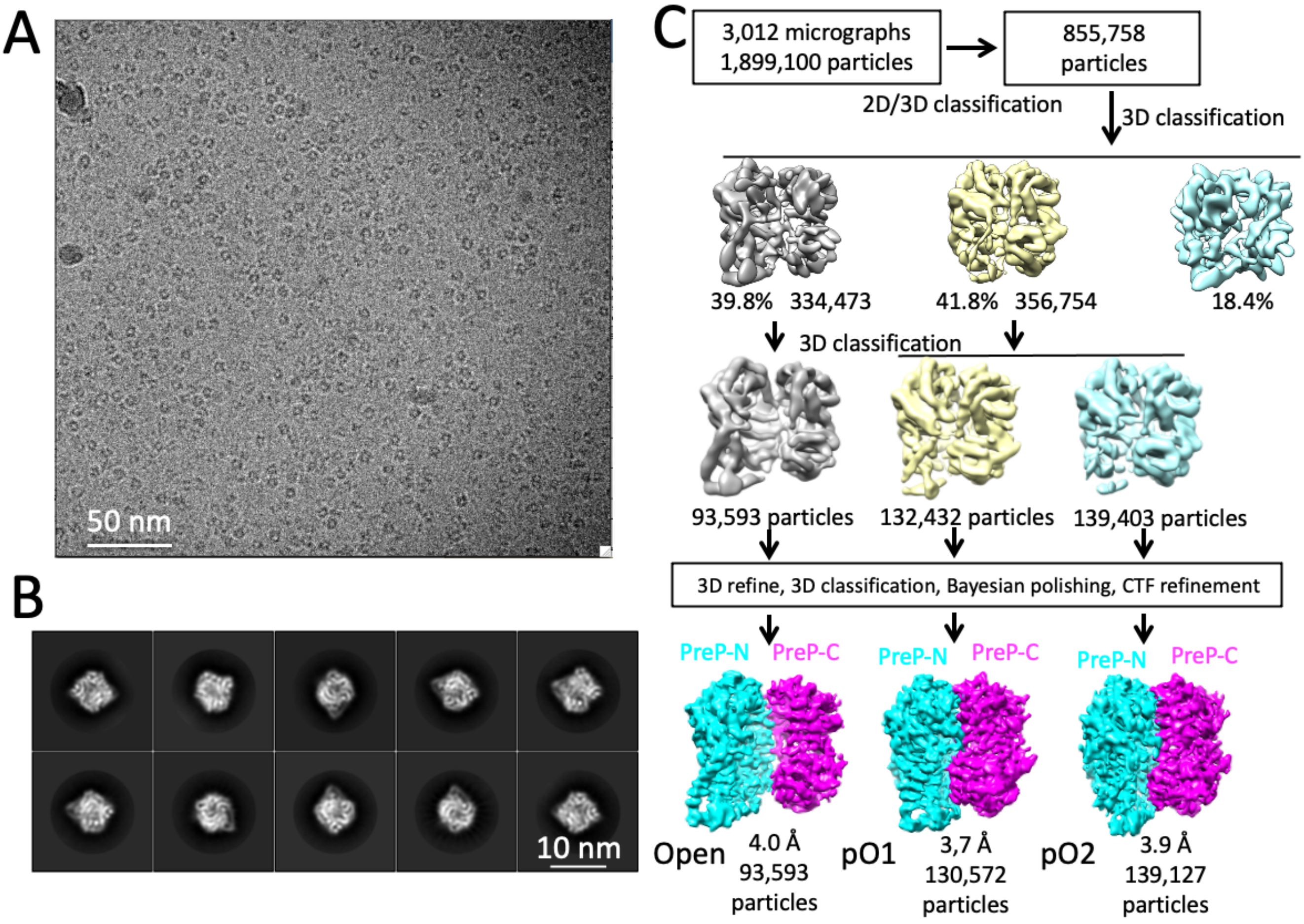

Supplementary Figure 7 CryoEM analysis of apo-PreP using grid prepared by Chameleon. **A**. CryoEM micrograph for apo-PreP prepared via Chameleon. Representative of 3,012 micrographs collected. **B**. 2D classification of PreP. **C**. The workflow of 2D/3D classification.

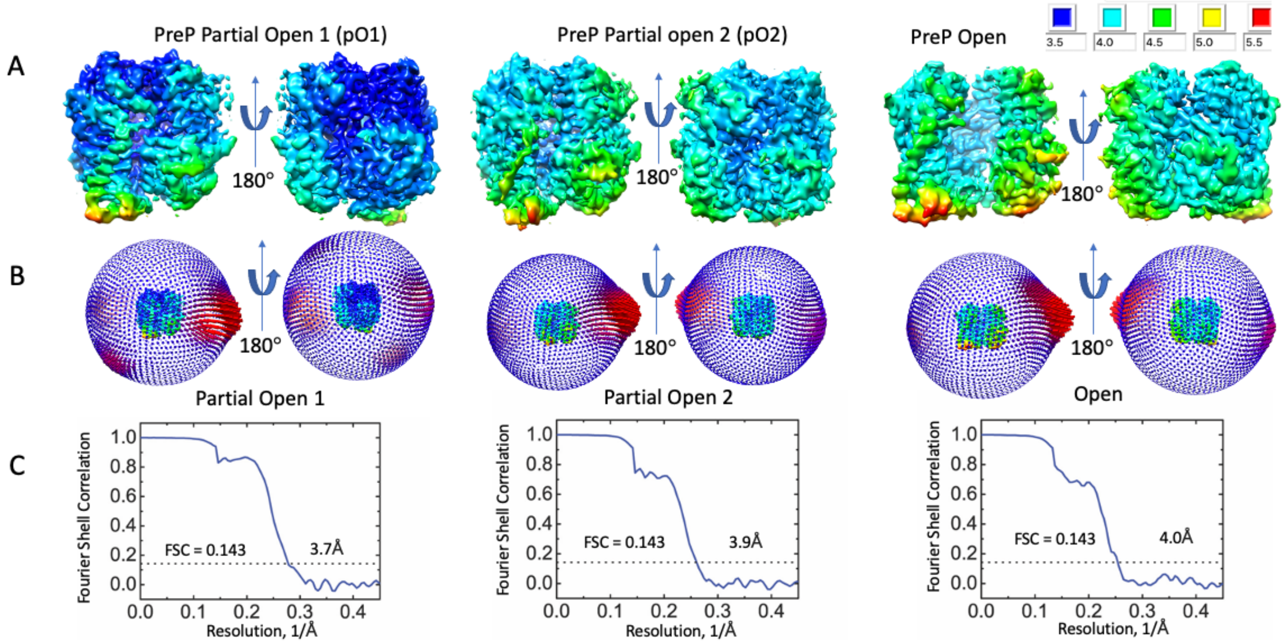

Supplementary Figure 8 Cryo-EM statistics of three apo-PreP structures, O, pO1, and pO2 using the grid prepared by Chameleon. (A) Final refined map colored according to local resolution generated from RELION3.0. (B) Angular distribution plots generated from RELION3.0. (C) FSC plot and resolution estimation using gold-standard 0.143 criterion generated from RELION3.0.

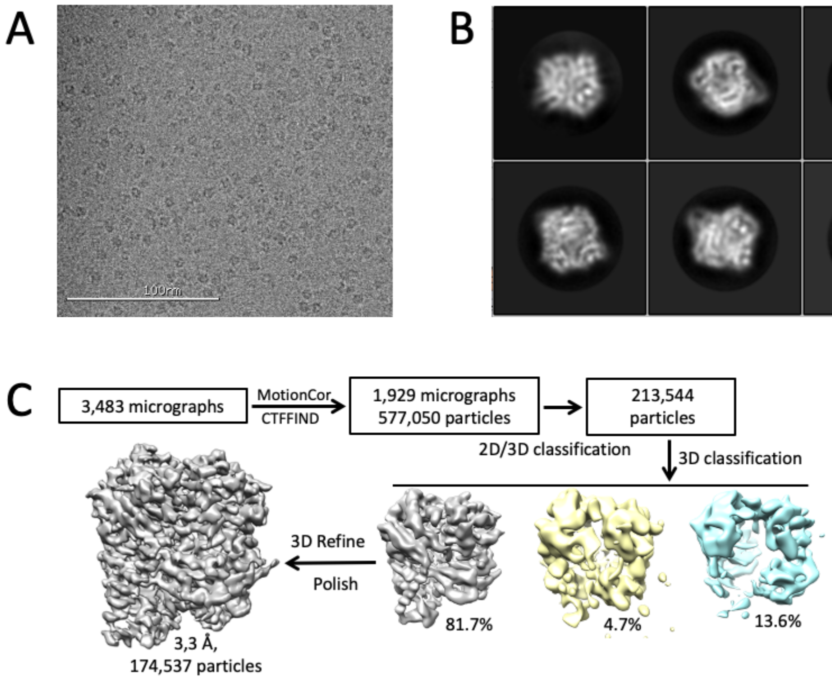

Supplementary Figure 9 CryoEM analysis of Aβ-bound PreP using grid prepared by Chameleon. A. CryoEM micrograph for PreP in the presence of Aβ representative of 3,483 collected micrographs. **B**. 2D classification of PreP. **C**. The workflow of 2D/3D classification.

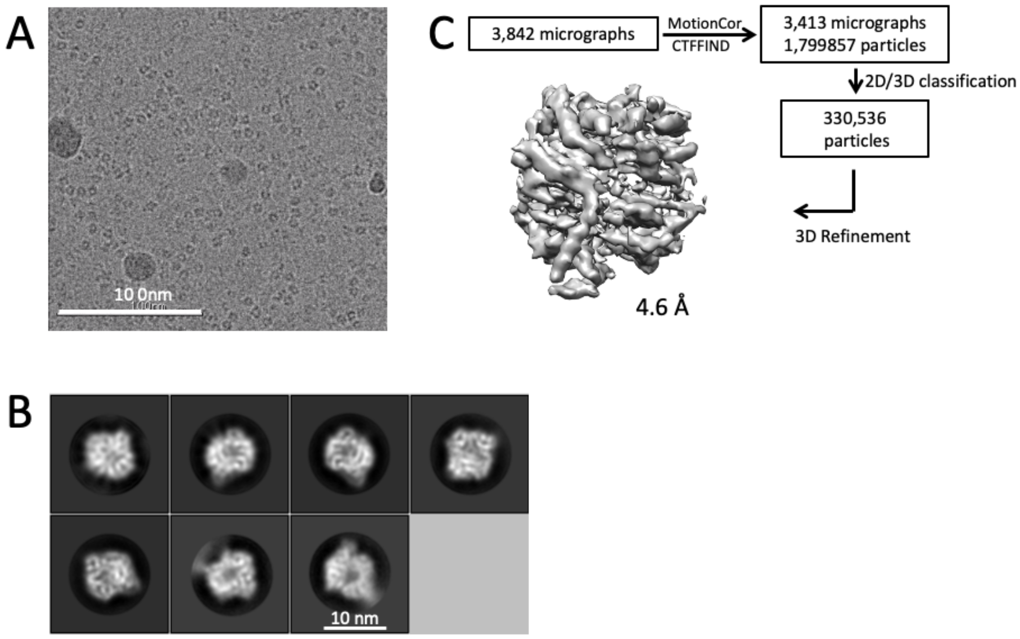

Supplementary Figure 10 CryoEM analysis of citrate-synthase presequence-bound PreP using grid prepared by Chameleon. A. CryoEM micrograph for PreP in the presence of citrate synthase presequence representative of 3,842 collected micrographs. **B**. 2D classification of PreP. **C**. The workflow of 2D/3D classification.

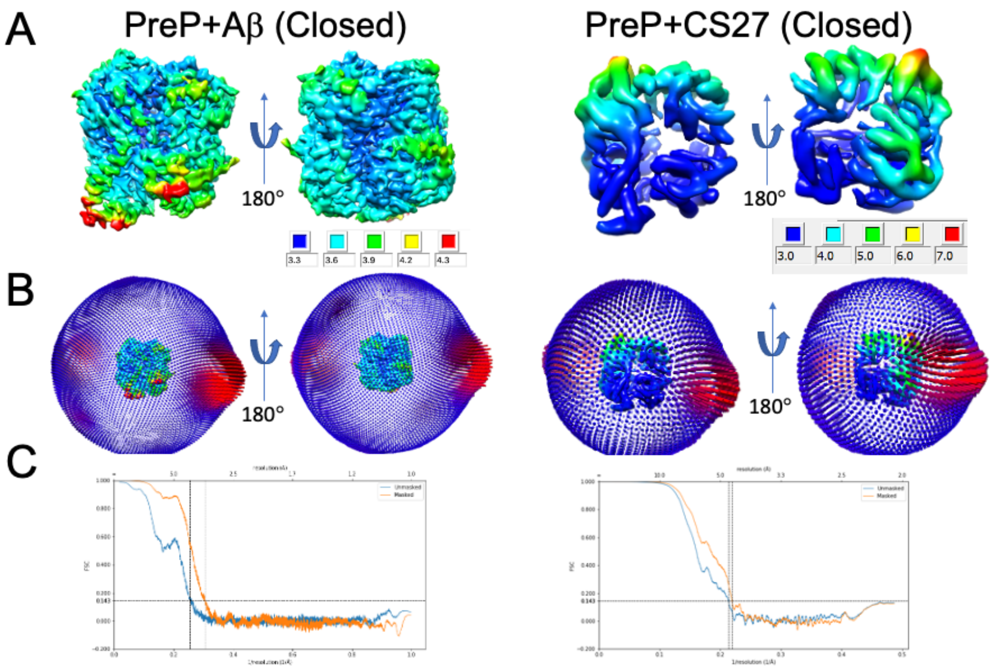

Supplementary Figure 11 Cryo-EM statistics of Aβ-bound and citrate synthase presequence-bound PreP structures. (A) Final refined map colored according to local resolution generated from RELION3.0. (B) Angular distribution plots generated from RELION3.0. (C) FSC plot and resolution estimation using gold-standard 0.143 criterion generated from Phenix.Mtriage.

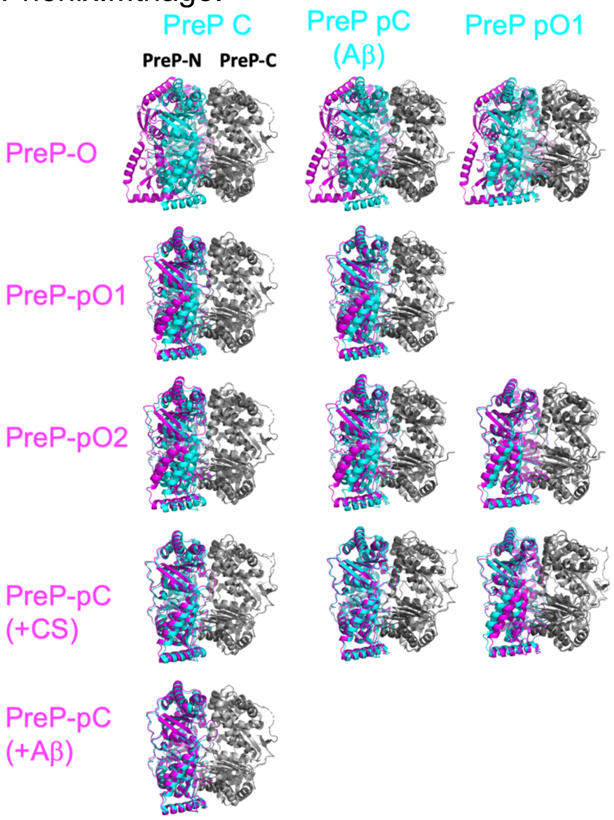

Supplementary Figure 12 Pairwise comparison of three apo-PreP cryoEM structures (open, pO1, and pO2), two substrate-bound cryoEM partial closed structures (Aβ-bound and citrate synthase (CS) presequence-bound PreP), and the closed state crystal structure of PreP (PreP-C). PreP N are colored either cyan or magenta of the indicated conformational states while PreP-C are colored in two different scales of grey. The comparison demonstrates that all six structures are different mostly by the rigid body motion between PreP-N and PreP-C.

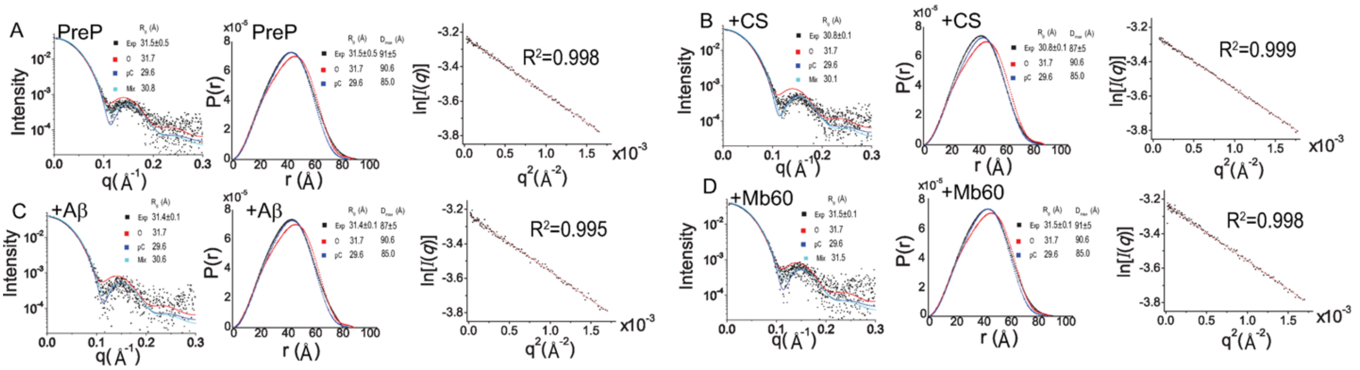

Supplementary Figure 13 SEC-SAXS analysis of PreP in the presence and absence of CS27, Aβ or MB60. SEC-SAXS scattering profiles and P(r) functions for (A) PreP alone, (B) PreP in the presence of citrate synthase presequence (CS27), (C) PreP in the presence of Aβ, and (D) PreP in the presence of MB60. Scattering profiles for the pC, pO, and O states were calculated using from structures with pdb codes of 6XOV, 6XOS, and 6XOU, respectively. Prior to calculating the scattering profiles, missing residues in the structural models (and the His-tag) were added based on the AlphaFold structure of PreP (AF-Q5JRX3-F1). The calculated single state scattering profiles, in general, resulted in suboptimal fits to the experimental data, suggesting a degree of conformational heterogeneity (see text). We modeled the scattering of a heterogenous mixture by using OLIGOMER to calculate the best fit scattering profile from a mixture of experimentally determined structures (Mix) and also utilized ensemble optimization modeling (EOM) to calculate the best fit scattering profile from a mixture of theoretical PreP structures. Guinier plots and model fits for the experimental data, OLIGOMER mixtures, and EOM are shown for (A) PreP alone, (B) PreP with CS27, (C) PreP with Aβ, and (D) PreP with MB60. The state distribution for the mixed population models is indicated underneath the plots.

A

Ribbon Map of PreP

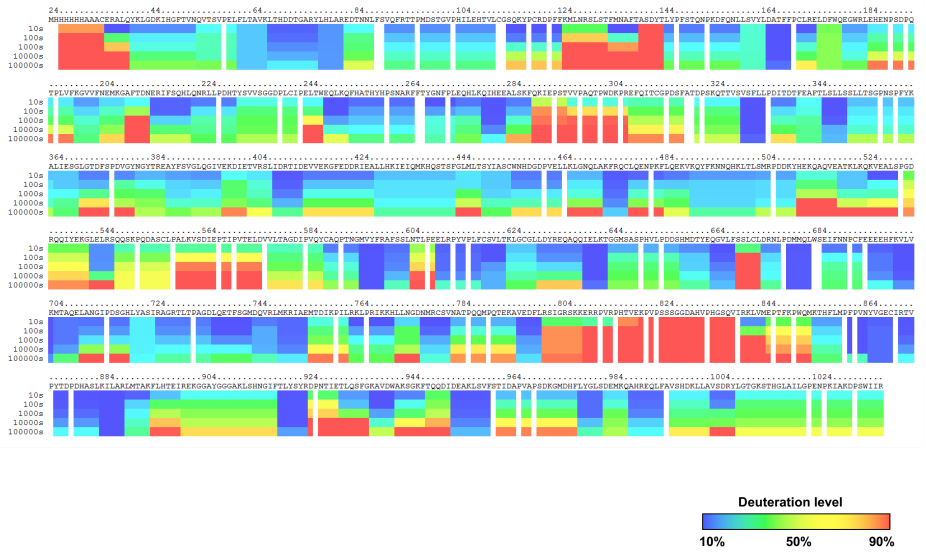

B

Ribbon Map of PreP+DMSO

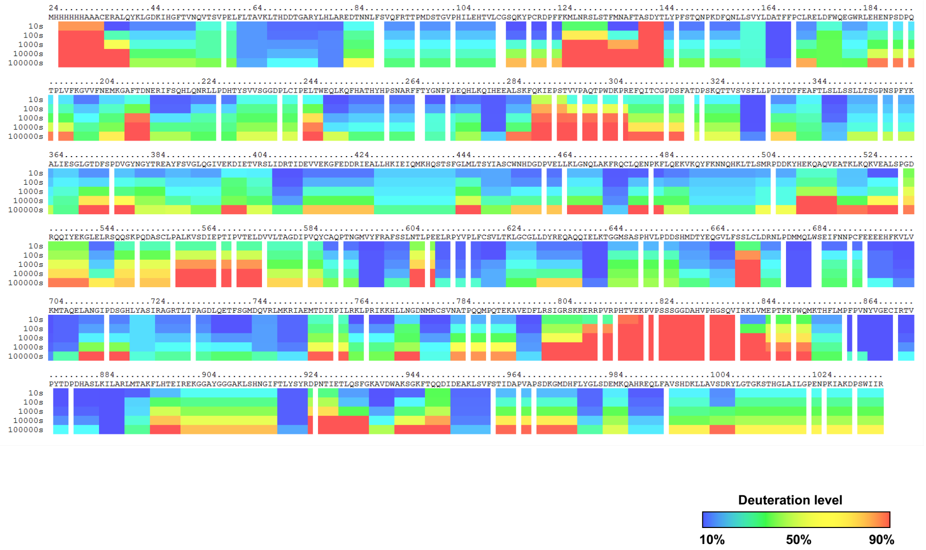

C

Ribbon Map of PreP+DMSO+MB60

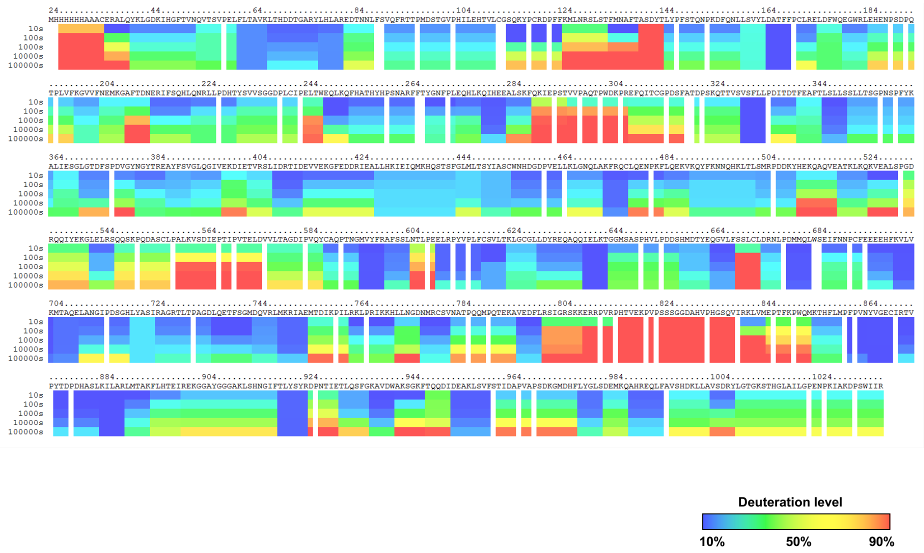

D

Ribbon Map of PreP

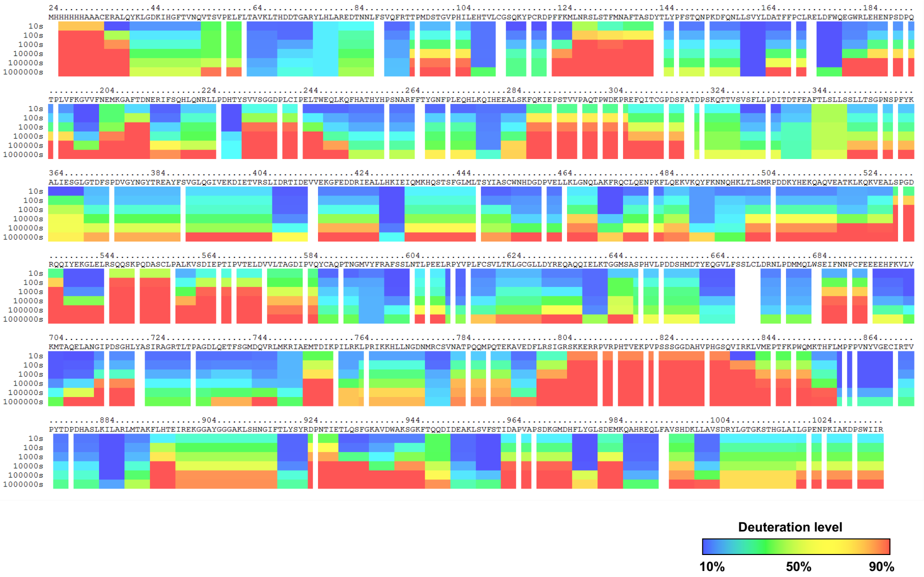

E

Ribbon Map of PreP+Aβ

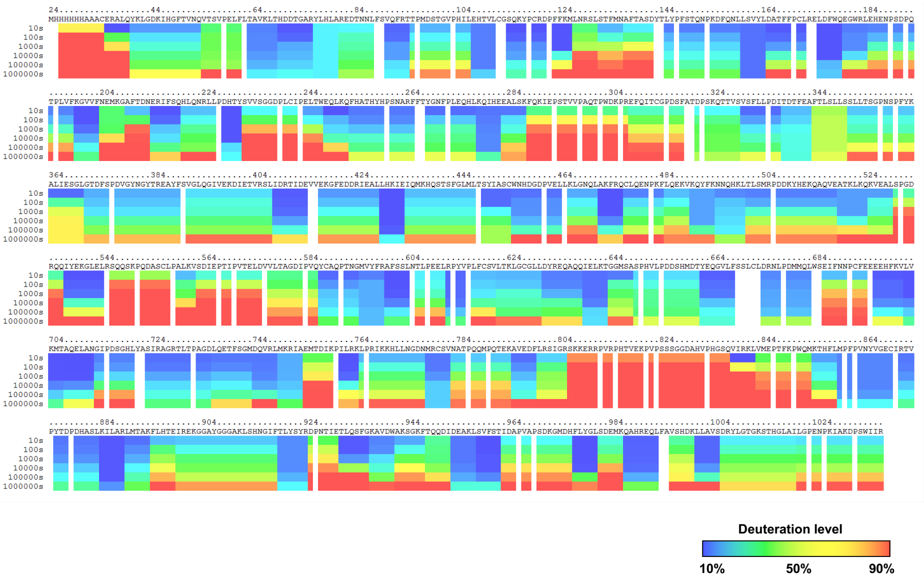

F

Ribbon Map of PreP

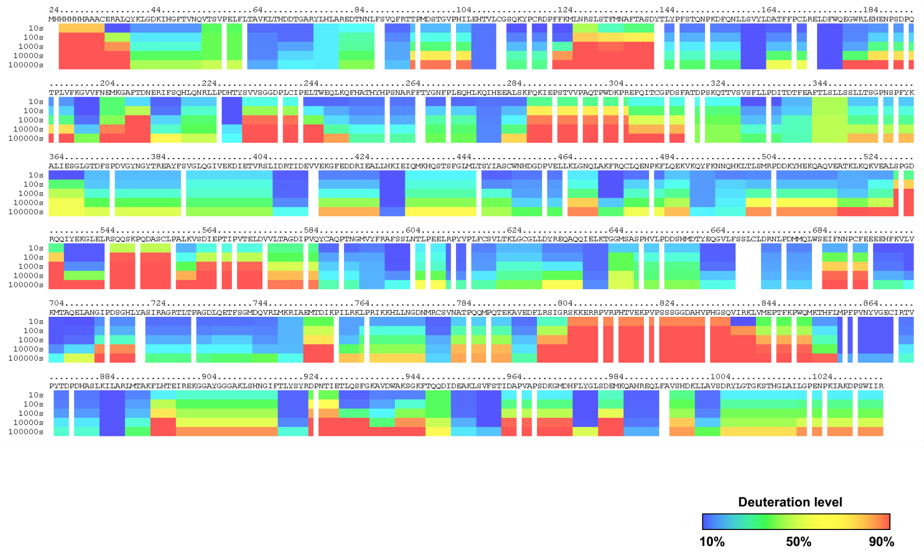

G

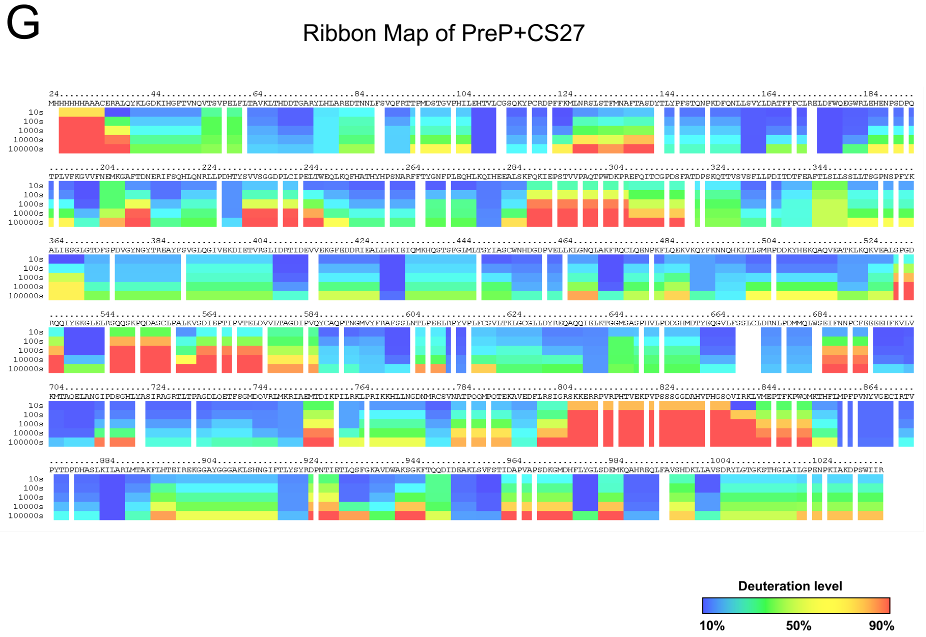

Supplementary Figure 14 Summary of hydrogen/deuterium exchange rates of PreP (A), in the absence (B) and presence (C) of MB60 with DMSO, or in the absence (D) and presence of substrate, amyloid beta 1-40 (Aβ) (E) and in the absence (F) and presence of the presequence of citrate synthase (CS27) (G). Deuteration levels of representative peptide fragments at various time points (from top to bottom: 10, 100, 1,000, 10,000, and 100,000 s at 0ºC are shown as a pseudo color scale. The percentages of deuteration levels of each peptide fragment at various time points are shown as a heat map color-coded from blue (<10%) to red (>90%), as indicated at the bottom right of the figure.

A

PreP+DMSO minus PreP

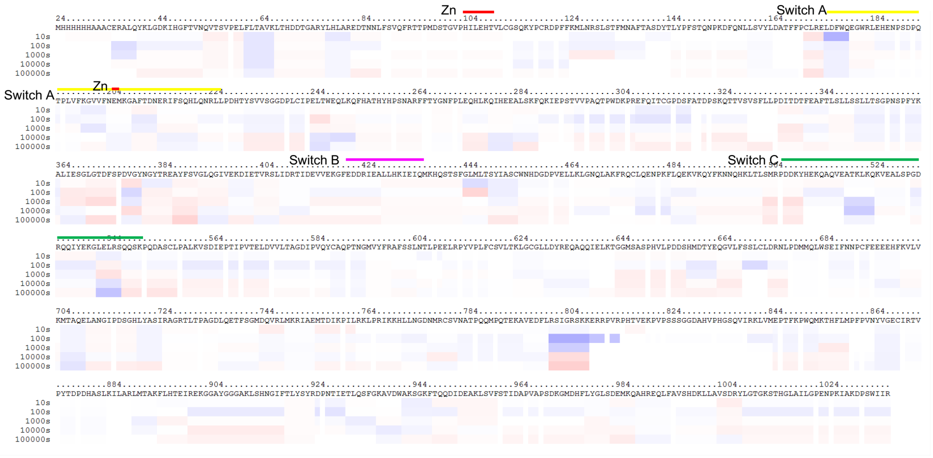

Blue suggests the regions which HD exchange became slower upon DMSO binding;
Red suggests the regions which HD exchange became faster upon DMSO binding.

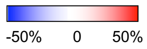

B

PreP+DMSO+MB60 minus PreP+DMSO

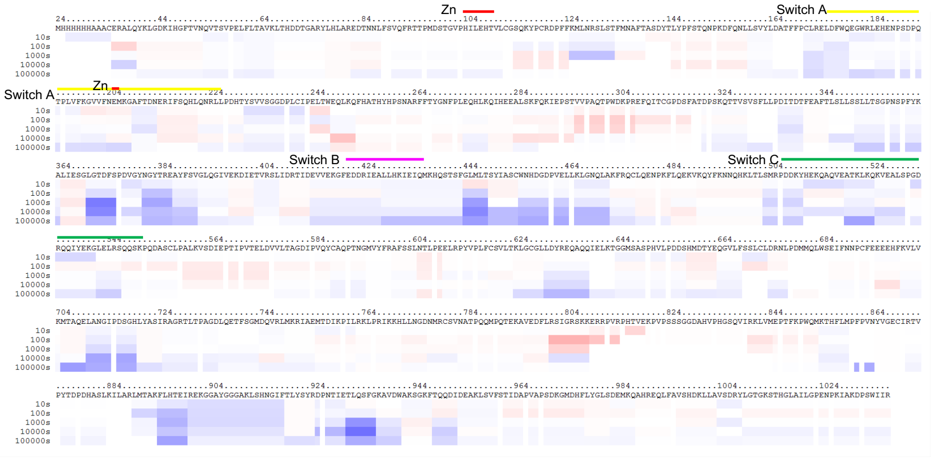

Blue suggests the regions which HD exchange became slower upon MB-60 binding;
Red suggests the regions which HD exchange became faster upon MB-60 binding.

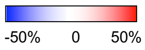

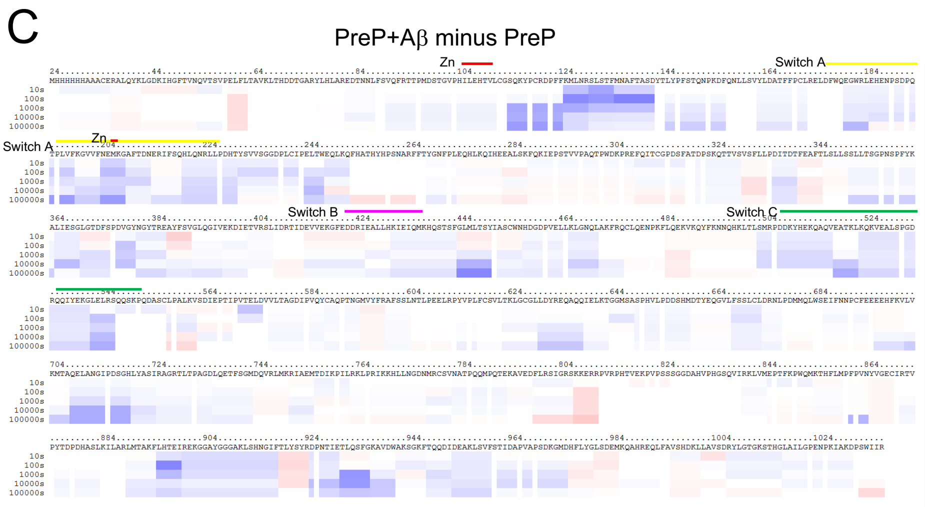

Blue suggests the regions which HD exchange became slower upon Aβ binding;
Red suggests the regions which HD exchange became faster upon Aβ binding.

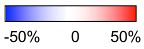

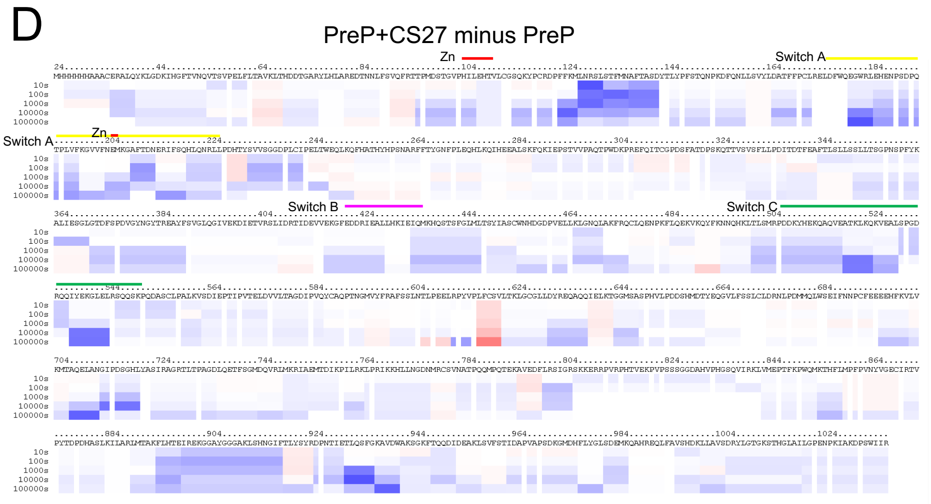

Blue suggests the regions which HD exchange became slower upon CS27 binding;
Red suggests the regions which HD exchange became faster upon CS27 binding.

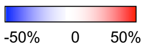

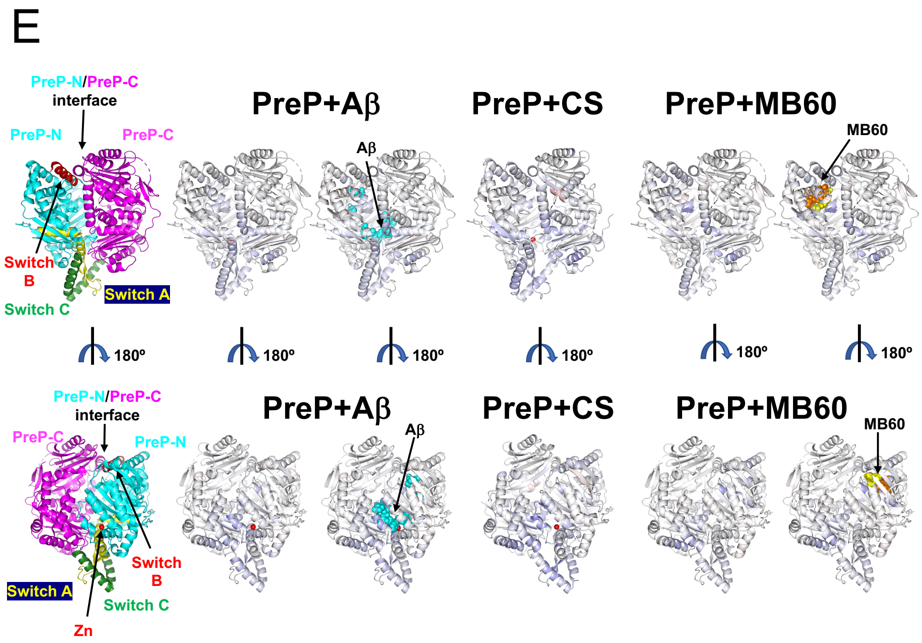

Figure 15 Changes in hydrogen/deuterium exchange rates of PreP. Changes in the rate of HDX induced by the binding of (A) DMSO, (B) MB60 (B), (C) amyloid beta (Aβ) and (D) the presequence of citrate synthase (CS27). Differences in deuteration levels between that with MB60 and that without MB60 at various time points (from top to bottom: 10, 100, 1,000, 10,000, 100,000 s) are shown in a color-coded bar ranging from blue (-50%) to red (+50%), as indicated at the bottom right of the figure. Key structural features are marked. (E) Map of the averaged change of HDX induced by Aβ, CS27, and MB60 onto the PreP structure. To avoid the obstruction by Aβ and MB60, PreP structures with the mapped HDX change are shown twice, one without Aβ or MB60 (left) and the other with Aβ or MB60 (right, pdb codes = 4nge or 4rpu, respectively).

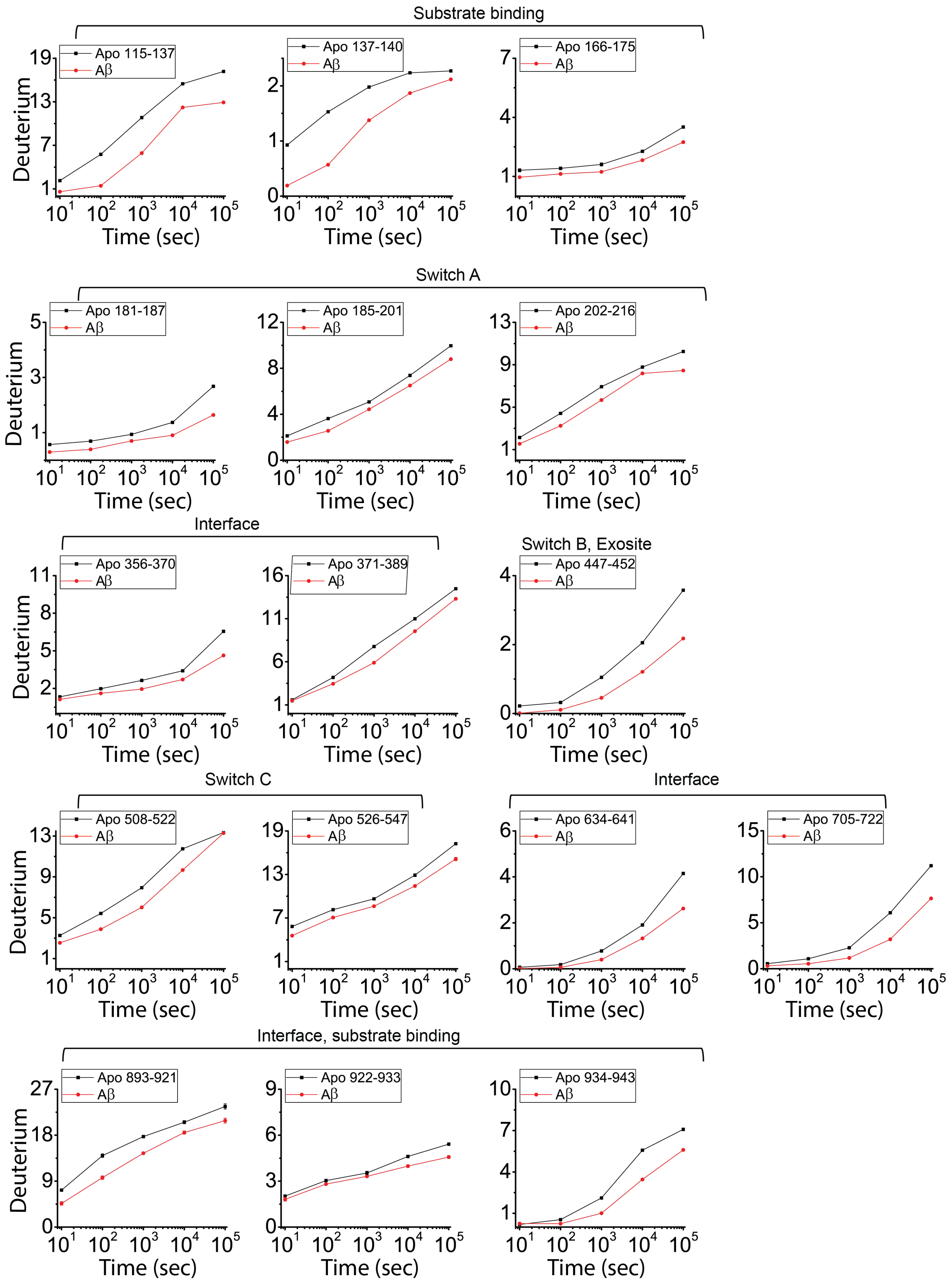

Supplementary Figure 16 Progression curve in hydrogen/deuterium exchange of PreP in the presence and absence of Aβ. Apo-PreP exchange shown in black, Aβ-PreP shown in red.

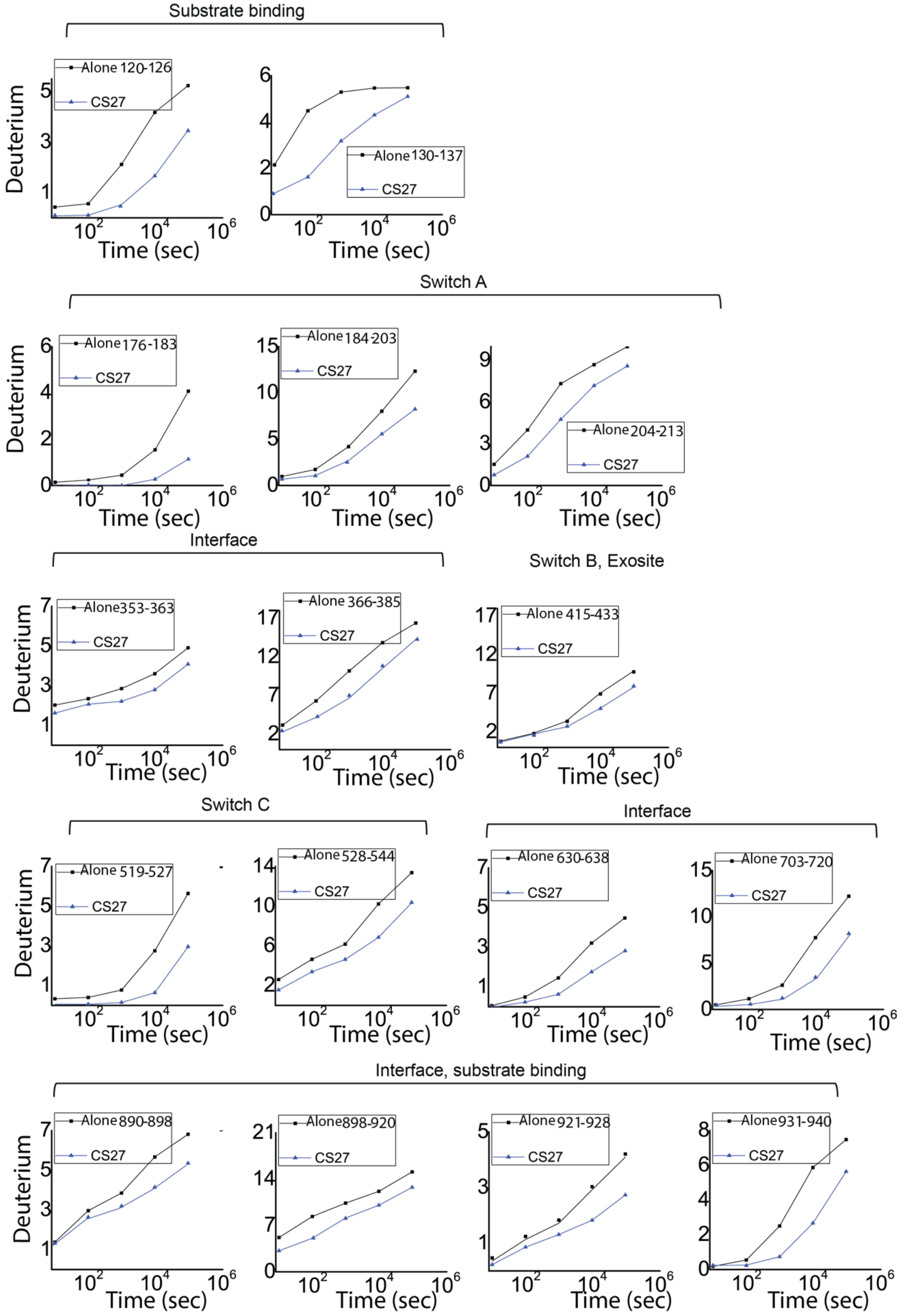

Supplementary Figure 17 Progression curve in hydrogen/deuterium exchange of PreP in the presence and absence of citrate synthase (CS27). Apo-PreP exchange shown in black, CS27-PreP shown in blue.

A

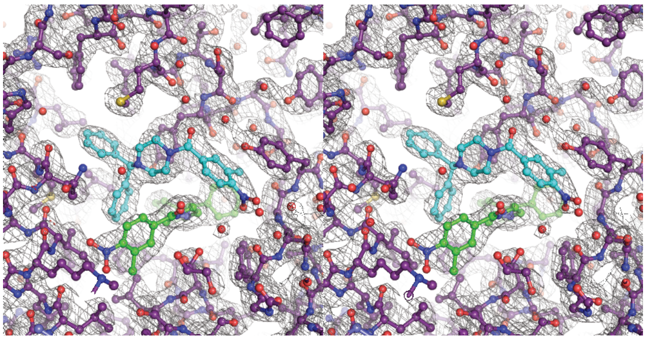

B

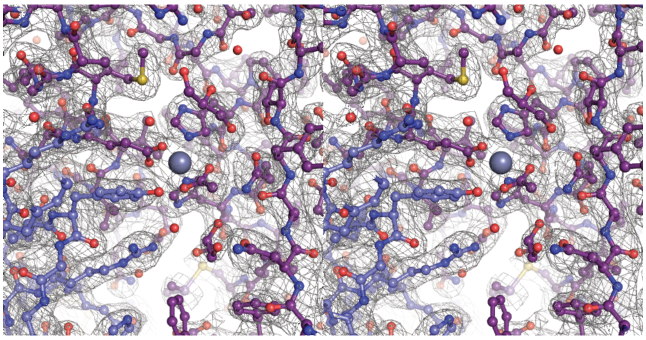

C

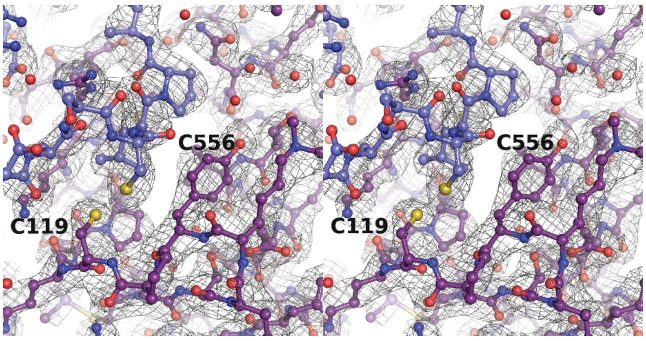

Supplementary Figure 18 Representative regions for the structures of PreP in complex with MB60 in stereoview. (A) MB60 binding site of PreP. (B) Zinc binding site of PreP. (C) Regions around cysteines 119 and 556. These two cysteines have been postulated to form the disulfide bond. 2mFo-DFc map (grey mesh) was contoured to 1σ. Walleye stereo view was prepared with PyMOL.

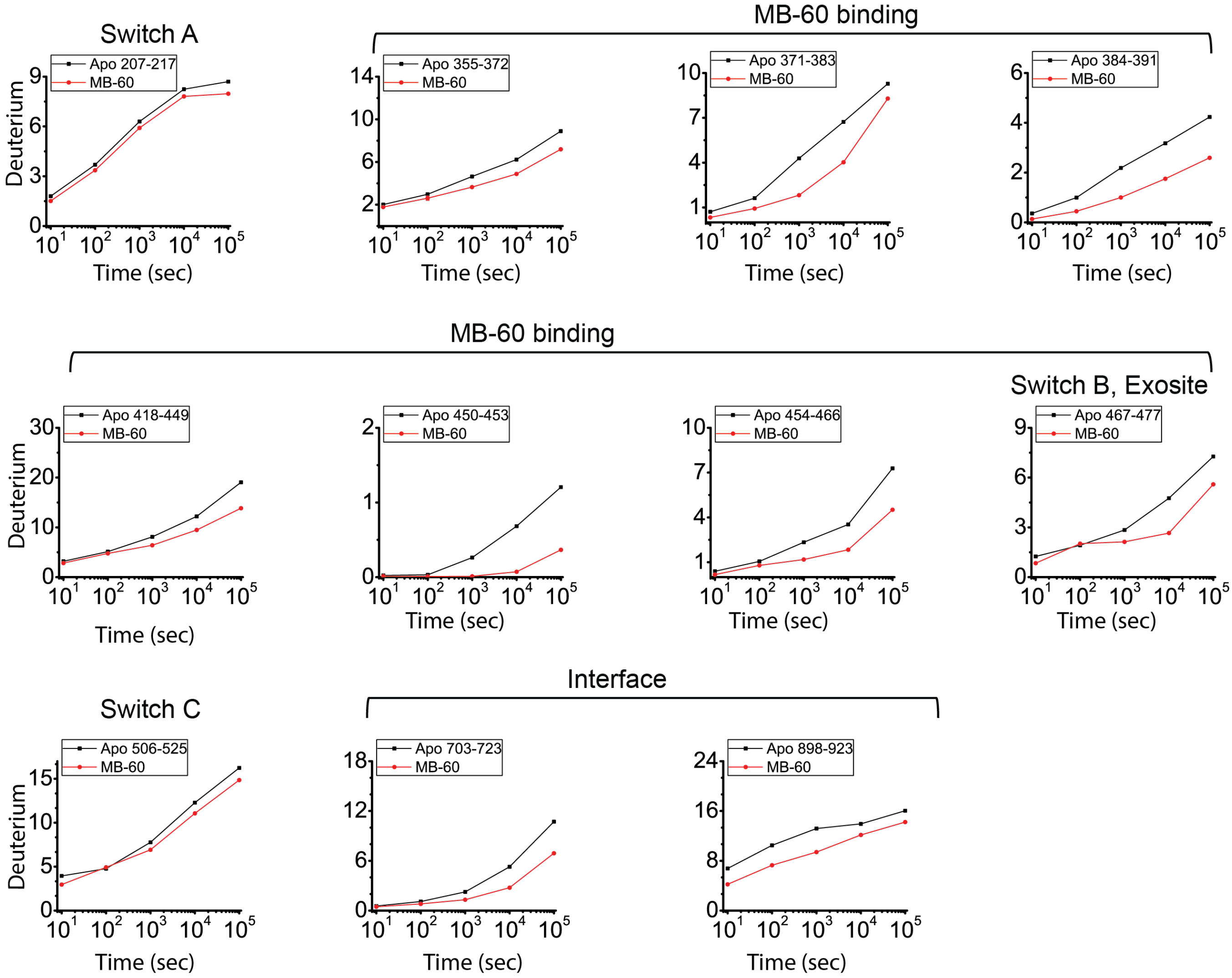

Supplementary Figure 19 Progression curve in hydrogen/deuterium exchange of PreP in the presence and absence of MB60. Apo-Prep exchange shown in black, MB60-PreP shown in red.

Supplementary Table 1 Comparison of seven PreP structures for domain motion and the interface between PreP-N and PreP-C.

	PreP N-C interface surface[1]			Distance[4] (PreP-N to C)	Angle[5] (D1-L-D4)	PDB code
	Buried surface	$\Delta^i G$ (kcal/mol)[2]	# of HB/ # of SB[3]			
PreP-O	1312 Å^2	-11.4	7/5	37.4 Å	83.3°	6XOU
PreP-pO1	2252 Å^2	-14.6	17/10	30.4 Å	63.8°	6XOS
PreP-pO2	1965 Å^2	-12.5	17/5	30.7 Å	64.1°	6XOT
PreP-pC(Aβ)	2776 Å^2	-18.0	23/11	29.3 Å	61.3°	6XOV
PreP-pC(CS27)	2370 Å^2	-23.0	12/2			6XOW
PreP-C	2606 Å^2	-20.6	24/12			4L3T
PreP-C(Aβ)	2820 Å^2	-18.7	27/15			4NGE

1. The protein interface surface analysis was done using EMBL-EBI PISA server (https://www.ebi.ac.uk/msd-srv/prot_int/cgi-bin/piserver). PreP-N includes aa 33-560 and PreP-C includes aa 561-1037.
2. $\Delta^i G$ indicates the solvation free energy gain upon formation of the interface, in kcal/M. The value is calculated as difference in total solvation energies of isolated and interfacing structures. Negative $\Delta^i G$ corresponds to hydrophobic interfaces, or positive protein affinity.
3. Numbers of hydrogen bond and salt bridge formed between PreP-N and PreP-C.
4. Distance between the center of mass of PreP-N and PreP-C.
5. Angle formed by the center of mass of PreP D1 domain, aa 562-564 (located at the boundary between PreP-N and PreP-C that does not undergo substantial conformational change), and D4 domain.

Supplementary table 2 SAXS data acquisition, sample details, data analysis, modelling fitting and software used.

**Sample details**	**PreP**	**PreP +CS**	**PreP +Aβ**	**PreP+MB60**
Organism	*Homo sapiens*	*Homo sapiens*	*Homo sapiens*	*Homo sapiens*
Source	*E. coli* expressed	CSBio (CS17324)	AnaSpec (AS-24236)	MolPort-001-620-747
UniProt sequence ID	Q5JRX3 (29-1037)	O75390 (1-27)	P05067 (688-711)	
Extinction coefficient [A_{280}, 0.1% (*w/v*)]	0.830	0.000	0.344	
Molecular mass from chemical composition (Da)	114,768	117,497	119,098	115,184
SEC-SAXS column, Superdex S200, 10/300G				
Loading concentration (mg ml^{-1})	12	10	10	11
Injection volume (µl)	200	250	250	240
Flow rate (ml min^{-1})	0.8	0.8	0.8	0.8
Solvent	20 mM Tris, pH 7.7, 100 mM NaCl with/without 20 mM EDTA			
**SAXS data collection parameters**				
Instrument/data processing	BioCAT/18ID beamline at Advanced Photon Source, Argonne National Laboratory (Chicago, USA)[1] using the photon counting PILATUS 3 1M			
Wavelength (Å)	1.0332			
Beam Size (µm^2) and Sample-to-detector distance	150(h) X 100(v) focused at detector, 3.6m			
q-measurement range (Å^{-1} or nm^{-1})	0.0043-0.3533			
Absolute scaling method	Glassy Carbon, NIST SRM 3600			
Basis for normalization to constant counts	To transmitted intensity by beam-stop counter			
Method for monitoring radiation damage, X-ray dose where relevant	Automated frame-by-frame comparison of relevant regions using CORMAP[2] implemented in BioXTAS RAW[3]			
Exposure time, number of exposures	0.5 s exposure time with a 1 s total exposure period (0.5 s on, 0.5 s off) of entire SEC elution			
Sample configuration including path length and flow rate where relevant	SEC-SAXS with sheath-flow cell[4], effective path length 0.542 mm. Size separation by an AKTA Pure with a Superdex 200 Increase 10/300 GL column.			
Sample temperature (ºC)	22			
**Software employed for SAS data reduction, analysis and interpretation**				
SAXS data reduction	Radial averaging; frame comparison, averaging, and subtraction done using BioXTAS RAW 2.0.3[3]			
Basic analyses: Guinier, P(r)	Guinier fit and M.W. using BioXTAS RAW, P(r) function using GNOM[5], RAW uses MoW and Vc M.W. methods[6,7]			
Mixed state modeling	OLIGOMER[8,9], EOM 2.0[10]			
**Structural parameters**	**PreP**	**PreP + CS27**	**PreP +Aβ**	**PreP+MB60**
Guinier Analysis				
I(0) (cm^{-1})	0.0397 ± 0.00004	0.039 ±0.00003	0.0395 ±0.00007	0.0396 ± 0.00005
R_g (Å)	31.56 ± 0.06	30.78 ± 0.06	31.37 ± 0.10	31.46 ± 0.08
q_{min} (Å^{-1})	0.0043	0.0043	0.0043	0.0043
Coefficient of correlation, R^2	0.9974	0.9985	0.9949	0.994
Experimental molecular weight (Da)	113104	119356	110667	112797
P(r) analysis				
I(0) (cm^{-1})	0.0395 ± 0.00004	0.0390 ± 0.00003	0.0392 ± 0.00005	0.0396 ± 0.00005
R_g (Å)	30.99 ± 0.04	30.24 ± 0.04	30.75 ± 0.05	30.97 ± 0.05
D_{max} (Å)	91	89	87	91
q-range (Å^{-1})	0.0043-0.3533	0.0043-0.3533	0.0043±0.3533	0.0043-0.3533
Total estimate from GNOM	0.9382	0.9247	0.8791	0.9379
Porod Volume	168000	165000	169000	165000
SASBDB IDs for data	**PreP**	**PreP+CS27**	**PreP+Aβ**	**PreP+MB60**
	SASDKK3	SASDKM3	SASDKN3	SASDKL3

Supplementary table 3 HDX-MS data acquisition, sample details, and data analysis.

Data Set	PreP	PreP-Aβ	PreP-CS27
HDX reaction details	150 mM Na_2Cl,<br>8.3 mM TRIS,<br>10mM EDTA,<br>pD_{read} = 7.2, 25 °C	150 mM Na_2Cl,<br>8.3 mM TRIS,<br>10mM EDTA,<br>pD_{read} = 7.2, 25 °C	150 mM Na_2Cl, 8.3 mM TRIS, 10mM EDTA,<br>pD_{read} = 7.2, 25 °C
HDX time course (min)	10, 100, 1000, 10000, 100000	10, 100, 1000, 10000, 100000	10, 100, 1000, 10000, 100000
HDX control samples	Maximally-labeled control (PreP protein)	Maximally-labeled control (PreP protein)	Maximally-labeled control (PreP protein)
Back-exchange (mean / IQR)	56.29% / 38.36%		
# of Peptides	463	463	463
Sequence coverage	100%	100%	100%
Average peptide length / Redundancy	18.4 / 8.4	18.4 / 8.4	18.4 / 8.4
Replicates (biological or technical)	2 (technical)	2 (technical)	1
Repeatability	0.132 (average standard deviation)	0.175 (average standard deviation)	0.0942 (average standard deviation)
Significant differences in HDX	10% D		

Data Set	PreP	PreP-DMSO	PreP-DMSO-MB60
HDX reaction details	150 mM Na_2Cl,<br>8.3 mM TRIS,<br>pD_{read} = 7.2, 25 °C	150 mM Na2Cl,<br>8.3 mM TRIS,<br>pDread = 7.2, 25 °C	150 mM Na2Cl,<br>8.3 mM TRIS,<br>pDread = 7.2, 25 °C
HDX time course (min)	10, 100, 1000, 10000, 100000	10, 100, 1000, 10000, 100000	10, 100, 1000, 10000, 100000
HDX control samples	Maximally-labeled control (PreP protein)	Maximally-labeled control (PreP protein)	Maximally-labeled control (PreP protein)
Back-exchange (mean / IQR)	56.34% / 37.67%		
# of Peptides	497	497	497
Sequence coverage	100%	100%	100%
Average peptide length / Redundancy	18.7 / 9.2	18.7 / 9.2	18.7 / 9.2
Replicates (biological or technical)	2 (technical)	2 (technical)	2 (technical)
Repeatability	0.309 (average standard deviation)	0.197 (average standard deviation)	0.209 (average standard deviation)
Significant differences in HDX	10% D		

Supplementary References:

1 Fischetti, R. *et al.* The BioCAT undulator beamline 18ID: a facility for biological non-crystalline diffraction and X-ray absorption spectroscopy at the Advanced Photon Source. *Journal of synchrotron radiation* **11**, 399-405 (2004).

2 Franke, D., Jeffries, C. M. & Svergun, D. I. Correlation Map, a goodness-of-fit test for one-dimensional X-ray scattering spectra. *Nat Methods* **12**, 419-422 (2015).

3 Hopkins, J. B., Gillilan, R. E. & Skou, S. BioXTAS RAW: improvements to a free open-source program for small-angle X-ray scattering data reduction and analysis. *Journal of Applied Crystallography* **50**, 1545-1553 (2017).

4 Kirby, N. *et al.* Improved radiation dose efficiency in solution SAXS using a sheath flow sample environment. *Acta Crystallographica Section D-Structural Biology* **72**, 1254-1266 (2016).

5 Svergun, D. I. Determination of the Regularization Parameter in Indirect-Transform Methods Using Perceptual Criteria. *J Appl Crystallogr* **25**, 495-503 (1992).

6 Rambo, R. P. & Tainer, J. A. Accurate assessment of mass, models and resolution by small-angle scattering. *Nature* **496**, 477-481 (2013).

7 Piiadov, V., de Araujo, E. A., Neto, M. O., Craievich, A. F. & Polikarpov, I. SAXSMoW 2.0: Online calculator of the molecular weight of proteins in dilute solution from experimental SAXS data measured on a relative scale. *Protein Science* **28**, 454-463 (2019).

8 Bernado, P., Mylonas, E., Petoukhov, M. V., Blackledge, M. & Svergun, D. I. Structural characterization of flexible proteins using small-angle X-ray scattering. *J Am Chem Soc* **129**, 5656-5664 (2007).

9 Konarev, P. V., Volkov, V. V., Sokolova, A. V., Koch, M. H. J. & Svergun, D. I. PRIMUS: a Windows PC-based system for small-angle scattering data analysis. *J Appl Crystallogr* **36**, 1277-1282 (2003).

10 Tria, G., Mertens, H. D. T., Kachala, M. & Svergun, D. I. Advanced ensemble modelling of flexible macromolecules using X-ray solution scattering. *Iucrj* **2**, 207-217 (2015).
