# Supplementary material for: Structural basis for the mechanisms of human presequence protease conformational switch and substrate recognition
Source: Nat Commun. 2022 Apr 5;13:1833. doi: 10.1038/s41467-022-29322-4 (PMC8983764; doi:10.1038/s41467-022-29322-4)
Supplement: Supplementary file 3 — Description of Additional Supplementary Files [file 41467_2022_29322_MOESM3_ESM.docx]

**Description of Additional Supplementary Files**

File name: Supplemental Movie 1

Description: CryoET of Prep grid prepared using Vitrobot.

File name: Supplemental Movie 2

Description: Fit of 3D structures of apo-PreP to the Coulomb potential map.

File name: Supplemental Movie 3

Description: Fit of 3D structures of amyloid beta-bound PreP to the Coulomb potential map.

File name: Supplemental Movie 4

Description: Structural comparison of various conformational states of PreP.

File name: Supplemental Movie 5

Description: Detailed view of the conformational switch of human presequence protease.

File name: Supplemental Data 1

Description: The raw data for the uptake and SD of deuterium of PreP peptides in the presence and absence of amyloid β or CS27 peptide.

File name: Supplemental Data 2

Description: The raw data for the uptake and SD of deuterium of PreP peptides in PreP alone, PreP in addition of DMSO (control solvent), and in the presence of MB60 dissolved in DMSO.
