# Supplementary material for: Structural basis for the mechanisms of human presequence protease conformational switch and substrate recognition
Source: Nat Commun. 2022 Apr 5;13:1833. doi: 10.1038/s41467-022-29322-4 (PMC8983764; doi:10.1038/s41467-022-29322-4)
Supplement: Supplementary file 11 — Reporting Summary [file 41467_2022_29322_MOESM11_ESM.pdf]

nature portfolio

Corresponding author(s): Wei-Jen Tang

Last updated by author(s): January 26, 2022

Reporting Summary

Nature Portfolio wishes to improve the reproducibility of the work that we publish. This form provides structure for consistency and transparency in reporting. For further information on Nature Portfolio policies, see our Editorial Policies and the Editorial Policy Checklist.

Statistics

For all statistical analyses, confirm that the following items are present in the figure legend, table legend, main text, or Methods section.

n/a	Confirmed	
☐	☒	The exact sample size (*n*) for each experimental group/condition, given as a discrete number and unit of measurement
☐	☒	A statement on whether measurements were taken from distinct samples or whether the same sample was measured repeatedly
☒	☐	The statistical test(s) used AND whether they are one- or two-sided<br>*Only common tests should be described solely by name; describe more complex techniques in the Methods section.*
☒	☐	A description of all covariates tested
☒	☐	A description of any assumptions or corrections, such as tests of normality and adjustment for multiple comparisons
☒	☐	A full description of the statistical parameters including central tendency (e.g. means) or other basic estimates (e.g. regression coefficient) AND variation (e.g. standard deviation) or associated estimates of uncertainty (e.g. confidence intervals)
☒	☐	For null hypothesis testing, the test statistic (e.g. *F*, *t*, *r*) with confidence intervals, effect sizes, degrees of freedom and *P* value noted<br>*Give P values as exact values whenever suitable.*
☒	☐	For Bayesian analysis, information on the choice of priors and Markov chain Monte Carlo settings
☒	☐	For hierarchical and complex designs, identification of the appropriate level for tests and full reporting of outcomes
☐	☒	Estimates of effect sizes (e.g. Cohen's *d*, Pearson's *r*), indicating how they were calculated

Our web collection on statistics for biologists contains articles on many of the points above.

Software and code

Policy information about availability of computer code

Data collection	Leginon was used for cryoEM and cryoET data collection. BioXTAS RAW 2.0.3 and ATSAS 3.0.3 was used for SAXS data collection and reduction.
Data analysis	Relion3.0 was used for image processing, particle picking, and 2D and 3D classification. MotionCor2 1.2.1 was used for micrograph motion correction. GCTF 1.06 was used for CTF estimation. UCSF Chimera, Coot0.9.6, and Phenix were used for model building and refinement. Appion-protomo, Tomo3D, and Dynamo were used to cryoET data analysis. HKL3000 was used to for crystallographic data processing. Coot0.9.6, Phenix, and PYMOL were used for crystallographic data analysis. ATSAS 3.0.3, PRIMUS3.0.3, Gnom4.6, crysol2.8.4, Oligomer7.1, BioXtas Raw 2.1.0, RANCH 2.1, and GAJOE 2.1 were used for SAXS data analysis. BioXtas Raw 2.0.3 was used for SAXS data collection. HDEXaminer v2.5.1 was used for HDX-MS data analysis.

For manuscripts utilizing custom algorithms or software that are central to the research but not yet described in published literature, software must be made available to editors and reviewers. We strongly encourage code deposition in a community repository (e.g. GitHub). See the Nature Portfolio guidelines for submitting code & software for further information.

Data

Policy information about availability of data

All manuscripts must include a data availability statement. This statement should provide the following information, where applicable:

- Accession codes, unique identifiers, or web links for publicly available datasets
- A description of any restrictions on data availability
- For clinical datasets or third party data, please ensure that the statement adheres to our policy

Structure factor amplitudes and coordinates for the crystal structures of MB-60 bound PreP and A-bound PreP are deposited in the Protein Data Bank under

accession number 4RPU [http://doi.org/10.2210/pdb4RPU/pdb] and 4NGE [http://doi.org/10.2210/pdb4NGE/pdb], respectively. The 3D cryoEM density maps generated in this study is deposited in the Electron Microscopy Data Bank under accession codes EMD-22278 [https://www.emdataresource.org/EMD-22278] (Apo-PreP pC1 state), EMD-22279 [https://www.emdataresource.org/EMD-22279] (Apo-PreP pC2 state), EMD-22280 [https://www.emdataresource.org/EMD-22280] (Apo-PreP open state), EMD-22281 [https://www.emdataresource.org/EMD-22281] (A-bound PreP), and EMD-22282 [https://www.emdataresource.org/EMD-22282] (CS27-bound PreP). The atomic coordinates is deposited in the Protein Data Bank under accession numbers 6XOS [http://doi.org/10.2210/pdb6XOS/pdb] (Apo-PreP pC1 state), 6XOT [http://doi.org/10.2210/pdb6XOT/pdb] (Apo-PreP pC2 state), 6XOU [http://doi.org/10.2210/pdb6XOU/pdb] (Apo-PreP open state), 6XOV [http://doi.org/10.2210/pdb6XOV/pdb] (A-bound PreP), and 6XOW [http://doi.org/10.2210/pdb6XOW/pdb] (CS27-bound PreP). EM data in the form of unprocessed micrographs is deposited in the Electron Microscopy Public Image Archive (EMPIAR) under accession number EMPIAR-10937 [https://www.ebi.ac.uk/empiar/EMPIAR-10937/]. The tomogram shown in the figures is deposited to the EMD with the accession number EMD-25921 [https://www.emdataresource.org/EMD-25921]. Raw tomography data is deposited to EMPIAR with the accession number EMPIAR-10929 [https://www.ebi.ac.uk/empiar/EMPIAR-10929/]. The HDX-MS data is deposited in ProteomeXchange under the accession number PXD029542 [http://proteomecentral.proteomexchange.org/cgi/GetDataset?ID=PXD029542]. SAXS data is deposited in the Small Angle Scattering Biological Data Bank (SASBDB) under accession codes SAS2721 [https://www.sasbdb.org/data/SAS2721] (Apo-PreP), SAS2722 [https://www.sasbdb.org/data/SAS2722/] (MB60-PreP), SAS2723 [https://www.sasbdb.org/data/SAS2723/] (CS27-PreP), and SAS2724 [https://www.sasbdb.org/data/SAS2724/] (A-PreP). Source data are provided with this paper. Source data are provided with this paper.

Field-specific reporting

Please select the one below that is the best fit for your research. If you are not sure, read the appropriate sections before making your selection.

☒ Life sciences ☐ Behavioural & social sciences ☐ Ecological, evolutionary & environmental sciences

For a reference copy of the document with all sections, see nature.com/documents/nr-reporting-summary-flat.pdf

Life sciences study design

All studies must disclose on these points even when the disclosure is negative.

Sample size	Images for single particle cryoEM, macromolecular crystallography, SEC-SAXS and HDX-MS were collected for the maximal resolution when data collection from national faculties allowed. For cryoEM, datasets consisted of thousands of micrographs, from which millions of particles were obtained. Specific dataset statistics are available in figures S3, 7, 9, 10. For CryoET, 80 tilt series were collected across two datasets, from this data 7 tomograms were reconstructed from the best tilt series. For crystallography, dataset consisted of 119,317 unique reflections resulting in a completeness of 99.9% and average multiplicity of 3.5. For SEC-SAXS, the dataset detail is listed in supplementary table 2. For HDX-MS, the largest number of peptides were selected for HDXMS analysis to increase the resolution of the data.
Data exclusions	Data were not excluded from analysis.
Replication	Experimental replications were an intrinsic nature for the data collection and processing of cryoEM, crystallography, and SEC-SAXS. For CryoEM, data was refined as independent half-sets and correlation between half-sets was used to determine resolution cutoff following field conventions. For macromolecular crystallography, inherent measures of replication are listed in table 2 following field conventions. Of note are the redundancy of 3.5 and the Rwork/Rfree of 0.176/0.208. For SEC-SAXS, see the experimental details in supplementary table 2. HDXMS data were collected with n=2 for key experiments, and all repeated experiments were successful.
Randomization	Randomization for data processing of cryoEM, cryoET, crystallography, SAXS, and HDXMS were not possible. When applicable, data was analyzed equally with no sub-sampling. Thus, there was no requirement for randomization. For cryoEM, angular distribution indicates a sphericity >0.8, demonstrating sufficient sampling of random orientations to generate a 3D structure free of orientation bias. Ab initio initial models were used to prevent the introduction of model-centric bias. For crystallography, covariates were controlled following standard crystallographic practices as indicated by the Rwork/Rfree values. For SAXS analysis, covariates are controlled by incorporation of SEC directly upstream of SAXS data collection, ensuring homogenous, monodispersed sample conditions. For HDX-MS, all collected peptides were analyzed by HDXMS and no randomization is required.
Blinding	Blinding was not possible for cryoEM, cryoET, crystallography, SAXS, and HDX0MS studies as either experimental conditions were necessary for data analysis or experimental conditions were evident from the data . When applicable, quantifications were performed using pipeline applied equally to all conditions and replicates for a given probe.

Behavioural & social sciences study design

All studies must disclose on these points even when the disclosure is negative.

Study description	*Briefly describe the study type including whether data are quantitative, qualitative, or mixed-methods (e.g. qualitative cross-sectional, quantitative experimental, mixed-methods case study).*
Research sample	*State the research sample (e.g. Harvard university undergraduates, villagers in rural India) and provide relevant demographic information (e.g. age, sex) and indicate whether the sample is representative. Provide a rationale for the study sample chosen. For studies involving existing datasets, please describe the dataset and source.*
Sampling strategy	*Describe the sampling procedure (e.g. random, snowball, stratified, convenience). Describe the statistical methods that were used to predetermine sample size OR if no sample-size calculation was performed, describe how sample sizes were chosen and provide a rationale for why these sample sizes are sufficient. For qualitative data, please indicate whether data saturation was considered, and what criteria were used to decide that no further sampling was needed.*

Data collection	*Provide details about the data collection procedure, including the instruments or devices used to record the data (e.g. pen and paper, computer, eye tracker, video or audio equipment) whether anyone was present besides the participant(s) and the researcher, and whether the researcher was blind to experimental condition and/or the study hypothesis during data collection.*
Timing	*Indicate the start and stop dates of data collection. If there is a gap between collection periods, state the dates for each sample cohort.*
Data exclusions	*If no data were excluded from the analyses, state so OR if data were excluded, provide the exact number of exclusions and the rationale behind them, indicating whether exclusion criteria were pre-established.*
Non-participation	*State how many participants dropped out/declined participation and the reason(s) given OR provide response rate OR state that no participants dropped out/declined participation.*
Randomization	*If participants were not allocated into experimental groups, state so OR describe how participants were allocated to groups, and if allocation was not random, describe how covariates were controlled.*

Ecological, evolutionary & environmental sciences study design

All studies must disclose on these points even when the disclosure is negative.

Study description	*Briefly describe the study. For quantitative data include treatment factors and interactions, design structure (e.g. factorial, nested, hierarchical), nature and number of experimental units and replicates.*
Research sample	*Describe the research sample (e.g. a group of tagged Passer domesticus, all Stenocereus thurberi within Organ Pipe Cactus National Monument), and provide a rationale for the sample choice. When relevant, describe the organism taxa, source, sex, age range and any manipulations. State what population the sample is meant to represent when applicable. For studies involving existing datasets, describe the data and its source.*
Sampling strategy	*Note the sampling procedure. Describe the statistical methods that were used to predetermine sample size OR if no sample-size calculation was performed, describe how sample sizes were chosen and provide a rationale for why these sample sizes are sufficient.*
Data collection	*Describe the data collection procedure, including who recorded the data and how.*
Timing and spatial scale	*Indicate the start and stop dates of data collection, noting the frequency and periodicity of sampling and providing a rationale for these choices. If there is a gap between collection periods, state the dates for each sample cohort. Specify the spatial scale from which the data are taken*
Data exclusions	*If no data were excluded from the analyses, state so OR if data were excluded, describe the exclusions and the rationale behind them, indicating whether exclusion criteria were pre-established.*
Reproducibility	*Describe the measures taken to verify the reproducibility of experimental findings. For each experiment, note whether any attempts to repeat the experiment failed OR state that all attempts to repeat the experiment were successful.*
Randomization	*Describe how samples/organisms/participants were allocated into groups. If allocation was not random, describe how covariates were controlled. If this is not relevant to your study, explain why.*
Blinding	*Describe the extent of blinding used during data acquisition and analysis. If blinding was not possible, describe why OR explain why blinding was not relevant to your study.*

Did the study involve field work? ☐ Yes ☒ No

Reporting for specific materials, systems and methods

We require information from authors about some types of materials, experimental systems and methods used in many studies. Here, indicate whether each material, system or method listed is relevant to your study. If you are not sure if a list item applies to your research, read the appropriate section before selecting a response.

Materials & experimental systems

n/a	Involved in the study	
☒	☐	Antibodies
☐	☒	Eukaryotic cell lines
☒	☐	Palaeontology and archaeology
☒	☐	Animals and other organisms
☒	☐	Human research participants
☒	☐	Clinical data
☒	☐	Dual use research of concern

Methods

n/a	Involved in the study	
☒	☐	ChIP-seq
☒	☐	Flow cytometry
☒	☐	MRI-based neuroimaging

Eukaryotic cell lines

Policy information about cell lines

Cell line source(s)	Leukemia K562 (human, ATCC CCL-243)
Authentication	None of the cell lines have been authenticated.
Mycoplasma contamination	Cell lines were not tested for mycoplasma contamination but no indication of contamination was observed.
Commonly misidentified lines (See ICLAC register)	No commonly misidentified cell lines were used.
